# Stress-related factors in the emergence of transient global amnesia with hippocampal lesions

**DOI:** 10.3389/fnbeh.2014.00287

**Published:** 2014-08-29

**Authors:** Juliane Döhring, Alexander Schmuck, Thorsten Bartsch

**Affiliations:** Department of Neurology, Memory Disorders and Plasticity Group, University Hospital Schleswig-Holstein, University of KielKiel, Germany

**Keywords:** amnesia, CA1, hippocampus, stress, transient global amnesia

## Abstract

The transient global amnesia (TGA) is a rare amnesic syndrome that is characterized by an acute onset episode of an anterograde and retrograde amnesia. Its origin is still debated, but there is evidence for psychological factors involved in TGA. In neuroimaging, selective lesions in the CA1 field of the hippocampus can be detected, a region that is particularly involved in the processing of memory, stress and emotion. The aim of this study was to assess the role of psychological stress in TGA by studying the prevalence of stress related precipitating events and individual stress-related personality profiles as well as coping strategies in patients. The hypothesis of a functional differentiation of the hippocampus in mnemonic and stress-related compartments was also evaluated. From all 113 patients, 18% (*n* = 24) patients experienced emotional and psychological stress episodes directly before the TGA. In a cohort of 21 acute patients, TGA patients tend to cope with stress less efficiently and less constructively than controls. Patients who experienced a stress related precipitant event exhibited a higher level of anxiety in comparison to non-stress patients and controls. However, there was no difference between the general experience of stress and the number of stress inducing life events. The majority of patients (73%) did show typical magnetic resonance imaging (MRI) lesions in the CA1 region of the hippocampal cornu ammonis. There was no clear association between stressful events, distribution of hippocampal CA1 lesions and behavioral patterns during the TGA. Disadvantageous coping strategies and an elevated anxiety level may increase the susceptibility to psychological stress which may facilitate the pathophysiological cascade in TGA. The findings suggest a role of emotional stress factors in the manifestation of TGA in a subgroup of patients. Stress may be one trigger involved in the emergence of transient lesions in the hippocampal CA1 region, which are thought to be the structural and functional correlate of TGA.

## Introduction

The transient global amnesia (TGA) is a rare amnestic syndrome, that is characterized by a sudden onset of a selective antero- and retrograde amnesia (Bartsch et al., 2010). In patients with a TGA, transient lesions confined to the CA1 field of the hippocampal cornu ammonis can be detected in high-resolution magnetic resonance imaging (MRI) 24–72 h after the amnestic phase (Bartsch et al., 2006, 2007). These MRI lesions can be considered the structural correlate of the amnestic deficit reflecting a transient perturbation of hippocampal function (Bartsch et al., 2006, 2010, 2011).

As the etiology of TGA remains enigmatic even 50 years after its first systematic clinical description, the incidence of events directly preceding a TGA has therefore attracted great interest (Fisher and Adams, 1964; Quinette et al., 2006; Bartsch and Deuschl, 2010). In the majority of patients, precipitating events encompassing physical, psychological and emotional factors can be observed. In recent years, especially the association with psychopathological factors has been elucidated (Merriam et al., 1992; Inzitari et al., 1997; Pantoni et al., 2005; Noël et al., 2007, 2008, 2011). These complementary studies suggest that certain personality traits might be relevant in TGA. Quinette et al. (2006) found an increased frequency of patients showing a psychological or emotional instability. Pantoni et al. found a higher prevalence of a personal or family history of psychiatric conditions and diseases or phobic traits in comparison with patients who have had a transient ischemic attack (TIA) or healthy controls (Pantoni et al., 2005). These studies also suggest that the occurrence of TGA might be associated with events involving a stress response, changes of bodily homeostasis and emotional state in persons susceptible to these factors (Quinette et al., 2006; Bartsch and Deuschl, 2010; Bartsch and Butler, 2013). The role of stress related personality factors in the etiology of TGA, however, has not been studied so far.

The hippocampus, and in particular the CA1 region, is critically involved in the formation of memory, but also in the neuroendocrine regulation of stress responses to emotional challenges including fear and anxiety (Howland and Wang, 2008; Joëls et al., 2008; Joëls, 2009). The hippocampal involvement in the regulation of stress is reciprocal: this structure influences the hypothalamic-pituitary-adrenal axis but is also affected by stress hormones, a mechanism that is thought to constitute a conceptual bridge to neuropsychiatric disorders (de Kloet et al., 2005; Wingenfeld and Wolf, 2014). The deleterious effects of stress and cortisol on the hippocampus are thought to lead to changes in synaptic plasticity, structural alterations and functional impairments in CA1 neurons (McEwen et al., 1968; de Kloet, 2012). With regard to the organization of CA1 networks, recent animal data suggest a differential organization of CA1 networks into functional compartments, such as the dorsal hippocampus performing cognitive functions and the ventral hippocampus subserving the processing of stress, emotion, and affect (Moser and Moser, 1998; Dong et al., 2009; Fanselow and Dong, 2010).

These considerations raise the possibility that the emergence of hippocampal CA1 lesions in the pathophysiological cascade of TGA is associated with stress related environmental factors, characteristic personality profiles and coping strategies, leading to an enhanced vulnerability of hippocampal function.

Therefore, our aim in this study was threefold: first, to study the prevalence of stress-related precipitating events in a cohort of 113 patients with a TGA. In a cohort of 21 acute TGA patients, secondly we analyzed individual stress related personality profiles and coping strategies by means of established questionnaires focusing on mental handling of stress, stressful life events of the two years preceding the attack and coping strategies. Further, considering a differential function-structure relationship of the human hippocampus in the processing of emotional stress and memory, we also analyzed the association of stress related factors with the behavior during TGA and the distribution of hippocampal lesions.

## Patients and methods

### Prevalence of stress-related precipitating events

Between 2003 and 2010 data of 113 TGA patients (65.4 ± 7.6 yrs, 62 females) presenting to our neurological emergency unit were collected. Only patients who fulfilled the Hodges and Warlow’s diagnostic criteria were included (Hodges and Warlow, 1990). The data comprised a characterization of precipitating events occurring directly before acute TGA onset. The precipitating events were divided into the following classes: 1. emotional stress, 2. physical effort, 3. acute pain, 4. water contact/temperature change, 5. sexual intercourse, 6. other factors and 7. unspecified, according to the classification of Quinette et al. (2006). The classification of a precipitating episode as stressful was based on the patients’ and the accompanying relatives’ evaluation of its intensity and valence as well as on the assessment and review of the episodes by the investigator. Only non-ambiguous episodes were classified as stress episodes. There were no incidents of precipitating psychological trauma. To further distinguish psychological stress factors from non-emotional factors such as physical factors, we dichotomized the precipitating events in emotional stress (visiting a grave, visit to a critically ill relative, emotional quarrel, big birthday celebration) and physical precipitating events (physical effort, pain, water contact, temperature change and sexual intercourse). With regard to a structure-function analysis of hippocampal lesion patterns and behavioral phenotypes, the patient’s behavior during TGA was also documented in grossly classified subdivisions of hyperactive (concerned, disquietness, restlessness), hypoactive (lethargy, quiet wakefulness) and normal mental states as judged by the accompanying relatives and the investigator. The study was approved by the Ethical Committee of the University of Kiel and participants gave informed consent.

### Neuroimaging

Standard whole-brain MRIs were performed on a 3T unit (Philips Intera Achieva) in 108 patients (95.6%) 24 to 72 h after onset of TGA symptoms when the detectability of hippocampal lesions is highest (Bartsch et al., 2007). Distributions of hippocampal CA1 lesions as seen on MRI were correlated with behavioral results (Bartsch et al., 2011). In patients, a standardized clinical routine MRI (T1- and T2-weighted turbo spin echo sequences and diffusion-weighted images with subsequent maps of the apparent diffusion coefficient (ADC), slice thickness 2 mm) was performed. The voxel size for the diffusion-weighted images was 1.67 × 2.12 × 3 mm and 0.51 × 0.65 × 2 mm for the T2-weighted images.

### Stress-related personality factors and life events

For a detailed study of stress-related personality profiles and coping strategies we recruited a cohort of 21 TGA patients (age: 69.7 ± 5.7 yrs, 9 males) with a recent amnestic episode, whose precipitating events could be clearly assigned to either one of the two categories (stress-related and physical). Twenty healthy, age-matched persons (age: 69.7 ± 6.6 yrs, 8 males) acted as controls.

### Assessment of stress-related personality factors and life events

We used four established self-rating questionnaires that measure several facets of stress including subjective and objective stress load, personality traits as well as coping strategies and mental handling of stress.

1. The *Perceived Stress Scale (PSS*; Cohen et al., 1983) is one of the most common psychological instruments for measuring the perception of stress. The PSS is an ordinal rating scale concerning the subjective evaluation of perceived stress. The sum score of its 10 items was used for the statistical analysis.
2. The *Stress coping scale* [Stressverarbeitungsfragebogen (SVF)] measures 20 different coping strategies in the context of time- and situation stable personal traits as coping strategies influence the handling of stress (Janke and Erdmann, 1997). Thus, every person has an individual spectrum of coping strategies leading to a variable handling of stress. The SVF is adapted from the instructions of the Ways of Coping Questionnaire (WCQ; Folkman and Lazarus, 1988). It comprises 114 items, which sum scores constitute the 20 subscales of coping strategies.
3. The *Social Readjustment Rating Scale* (SRRS; Holmes and Rahe, 1967) measures the objective experience of 43 specific critical personal life events including negative as well as positive (though stress inducing) emotional events of the two years preceding the episode. The subject has to mark all personally experienced events on a given list. The sum score is calculated from values that are assigned to each event representing its degree of severity. Thus, a high sum score is interpreted as a great extent of objective stressors. Critical live events pertain to stress in a broader sense. We supposed that vulnerability increases according to the experience of stressful life events.
4. The *Hospital Anxiety and Depression Scale (HADS-D)* consists of 14 Items accounting either for the anxiety or depression subscale. The sum scores of the subscales refer to the general mental state 1 week before the attack. The HADS-D extends the concept of stress as stress-related factors can be critically involved in the development and maintenance of depression and anxiety.

### Neuropsychological assessment

In the acute amnestic TGA phase (Study 2), declarative memory was tested using the Rey Auditory Verbal Learning Test (RAVLT) as well as visuoconstructive memory using the Rey-Osterrieth Complex Figure Test (ROCF). In the follow up, both domains were retested using parallel versions of the RAVLT and the ROCF. General cognitive assessment in the follow up encompassed an estimation of the intelligence level (Mehrfachwahl-Wortschatz-Intelligenztest, an equivalent to the National Adult Reading Test (NART)), word fluency (Regensburger Wortflüssigkeits-Test, RWT) and executive functions (Trail Making Test A and B).

### Neurological assessment

All patients had a standard neurological examination on admission. The characteristics and time course of the TGA episode was documented including events and factors precipitating the TGA episode. We registered the presence of cardiovascular and other risk factors including the history and prevalence of migraine using the classification criteria of the International Headache Society ((IHS) ICHD-II).

### Statistical analysis

The Statistical Package for the Social Sciences (version 17.0) was used for data analysis. Interrelations between variables were calculated by Pearson’s product- moment correlation and Spearman’s rank based correlations, respectively. Continuous variables were compared with independent Student *t* tests (TGA patients and healthy controls) or *t* tests for dependent samples, respectively (patients in the acute TGA state vs. follow up). The normal distribution of continuous variables was tested by Kolmogorov- Smirnov statistics. Interrelations between nominally scaled variables were calculated using contingency tables and the corresponding Chi-square independence tests. Significance of 2 × 2 contingency tables was determined by Fisher’s Exact test. The level of significance was set at 0.05. Data are quoted as mean ± standard deviation.

## Results

### Prevalence of stress-related precipitating events

**Figure 1:**
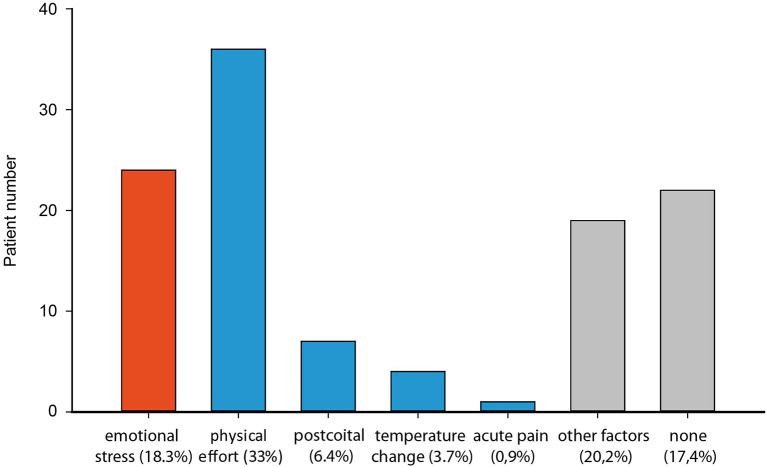
**Distribution of precipitating events of 113 patients with a TGA classified as emotional stress (red) and physical (blue) (physical effort, postcoital, temperature changes, acute pain) and others (grey)**.

The categorization of precipitating events immediately before TGA onset within a cohort of 113 TGA patients (mean age 65.4 ± 7.6 yrs; 45% males, 55% females) shows that 18% (*n* = 24) patients experienced emotional and psychological stress episodes (such as marital conflict, surgery of son at day of TGA and seeing grandchild for the first time; Figure 1). Forty-four percent of patients (*n* = 48) had a clear physical precipitant event, such as heavy lifting, cycling or swimming in a lake. This group of patients also includes those who experienced acute pain, sexual intercourse and temperature change immediately before TGA. The latter group was dominated by males, whereas significantly more women experienced an emotional stress-related precipitant, χ^2^ (Pearson) = 11.90, *df* = 1, Fisher’s exact test: *p* < 0.001. About a third of TGA patients experienced no designated precipitating situation or reported other factors, which could not be specified in the sense of both main categories.

#### Neuroimaging

The majority of patients (73%) did show typical MRI lesions in the CA1 region of the hippocampal cornu ammonis (unilateral lesion: 61.1%, bilateral lesions: 11.5%, and respectively ventral: 30.1%, intermediate: 20.4%, dorsal: 8.0%). In 23% of patients no lesions could be detected. In five patients, an MRI could not be performed. The type of precipitating event did not have an influence on the side of the hippocampal lesion (left/right), χ^2^ = 27.31, *df* = 20/*p* = 0.13, on the position within the longitudinal extension of the hippocampus, χ^2^ = 11.42, *df* = 16/*p* = 0.78, or on the total lesion load, χ^2^ (Pearson) = 0.63, *df* = 1, Fisher’s exact test: *p* = 0.71. There was no statistically significant relationship between laterality or location of CA1 features and risk factors.

#### Behavior during the acute TGA episode

The majority of amnesic patients (34.5%) displayed hyperactive behavior such as restlessness and concern during the acute TGA episode including the typical display of helplessness due to the retention span of a few minutes. Eighteen percent of patients showed hypoactive behavior. About a third could not be clearly classified within the described scheme and 11% of patients exhibited normal behavior. There was no influence of sex, χ^2^ = 3.79, *df* = 2/*p* = 0.15, age, Spearman’s ϱ= 0.03, *p* = 0.85, and precipitant events, χ^2^ = 1.25, *df* = 2/*p* = 0.54, on the acute behavioral patterns. Neither the side of the hippocampal lesion (left/right), χ^2^ = 8.58, *df* = 8/*p* = 0.38, nor the position within the longitudinal extension of the hippocampus (ventral, intermediate, dorsal), χ^2^ = 6.89, *df* = 8/*p* = 0.55, nor lesion load, χ^2^ = 1.54, *df* = 2/*p* = 0.46, exerted an influence on the acute behavior.

#### Risk factor profile

The prevalence of risk factors in the large cohort corresponds to the distribution within the healthy, age-matched population (Quinette et al., 2006; Enzinger et al., 2008; Romero et al., 2013; Mangla et al., 2014). This applies to transient ischemic attacks, arterial hypertension, coronary heart disease, cardiac arrhythmia, subcortical arteriosclerotic encephalopathy, preexisting insults, lacunar stroke and diabetes. The only exception was the apparently high proportion of migraine in the medical history (thirty-two persons = 28.3%) (Quinette et al., 2006; Lin et al., 2014).

### Stress related personality factors and life events in TGA

The results of the Stress coping scale revealed two significant differences between TGA patients and healthy controls: TGA patients tended to increased feelings of guilt, *t*_(48)_ = −2.30, *p* = 0.03, whereas controls downplayed their troubles by comparing with other people, *t*_(48)_ = 2.83, *p* = 0.01. No significant differences in the general experience of stress, measured by the sum score of the PSS could be detected between TGA patients and controls, *t*_(43)_ = 0.00, *p* = 1.00. Further, the SRRS revealed no disparity with a view to the number of stress inducing life events, *t*_(43)_ = 0.04, *p* = 0.97. The HADS-D did not show differences in the anxiety, *t*_(46)_ = −1.32, *p* = 0.19, and depression scores, *t*_(46)_ = −0.49, *p* = 0.63.

#### Personality factors and life events in patients with emotional stress and physical effort

For further analyses, the cohort of study 2 was dichotomized into two groups of precipitating emotional stress and physical events. The HADS-D revealed a significant higher anxiety level in the stress group, *t*_(26)_ = 3.42, *p* < 0.01. This also applies to the comparison to healthy controls, *t*_(30)_ = −2.22, *p* = 0.03. Furthermore, the Stress coping scale showed a significant difference in coping strategies: Persons who had an emotionally arousing precipitating event are more likely prone to downplay of their troubles, *t*_(26)_ = 2.23, *p* = 0.03, (comparison to healthy controls: *t*_(32)_ = 2.57, *p* = 0.15) and tended to use more medication, *t*_(26)_ = 1.99, *p* = 0.06. We neither could detect any significant influence of general subjective stress-experience (measured by the PSS, *t*_(26)_ = 0.87, *p* = 0.39), nor any influence of the objective amount of stressors (measured by the SRRS, *t*_(26)_ = 1.45, *p* = 0.16), on the type of precipitant events.

#### Neuropsychological assessments

**Figure 2:**
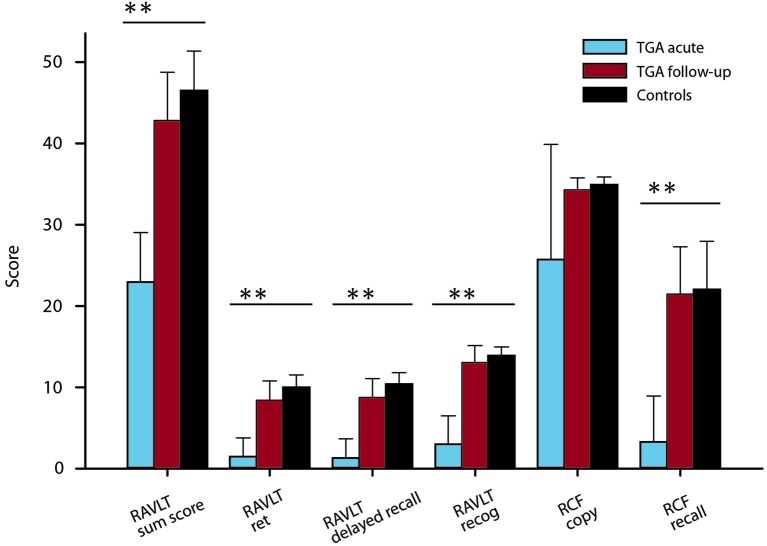
**Verbal and visuoconstructive memory results of the RAVLT and the Rey-Osterrieth complex figure of TGA patients (acute and follow-up) (mean ± SD)**. *P* values from t tests; **P < 0.01. Control values are from Bartsch et al. (2011).

During the acute phase, declarative memory of TGA patients (word pairs, measured by the RAVLT) was severely impaired (acute vs. follow up: *t*_(16)_ = −10.72, *p* < 0.01; Figure 2) compared to follow-up and control levels (Bartsch et al., 2011). Additionally, visuoconstructive memory was significantly affected during the attack, *t*_(18)_ = −9.59, *p* < 0.01. A comparison between patients with an emotional and patients with a physical precipitant did not detect any significant difference in auditory verbal learning, *t*_(15)_ = 1.03, *p* = 0.32, and visuoconstructive memory, *t*_(17)_ = −1.14, *p* = 0.27, during the acute phase. Executive functions, intelligence and word fluency tested during follow up were within the normal range. With regard to laterality, the majority of patients were right-handed (95%).

#### Distribution of hippocampal lesions

**Figure 3:**
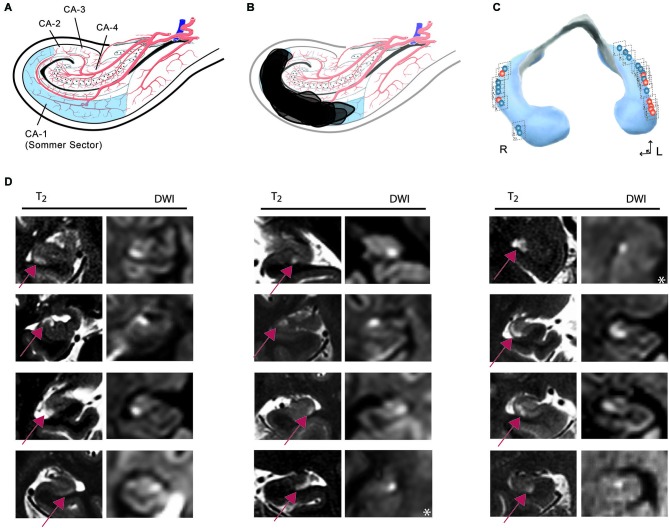
**(A)** Anatomical template showing a representative coronal slice of the hippocampal cornu ammonis indicating sectors after Lorente de Nó. **(B)** Synopsis of all DWI/T2 lesions transferred to an anatomical template of the cornu ammonis. **(C)** Distribution of lesions along the longitudinal axis of the hippocampus. Red lesions: stress-related precipitant episodes; blue: physical episodes. **(D)** Columns show representative coronal MRI images of the hippocampus [T2/Diffusion-weighted images (DWI)] illustrating that lesions were confined to the CA1 area of the cornu ammonis. In each CA1 lesion, signal changes in diffusion-weighted and corresponding T2-weighted imaging can be observed. The arrow illustrates the CA1 lesions in T2 imaging. *DWI image in an axial plane.

**Figure 4:**
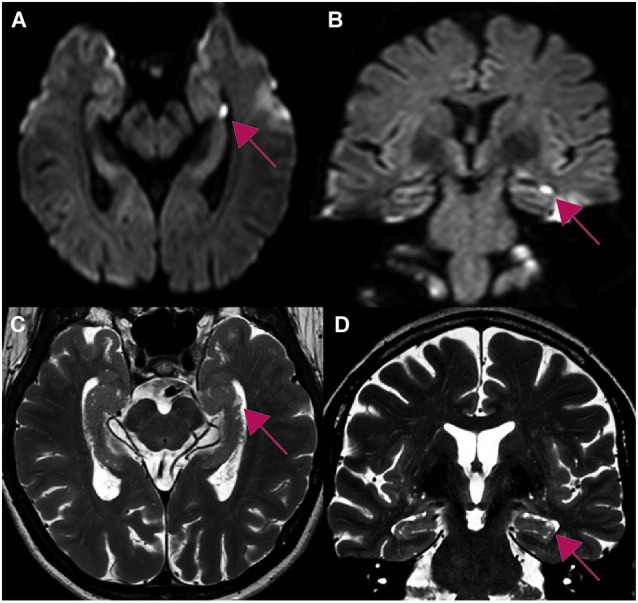
**Whole brain 3T MR-imaging of one patient showing a focal lesion in the head of the left hippocampus in CA1 (red arrow) after an emotional stressful event**. The lesion in the diffusion-weighted imaging **(A,B)** correlates with the lesion in T2-weighted sequences **(C,D)**. Table 1: Study 2: demographic data, precipitating events and distribution of hippocampal lesions. Ant, anterior; post, posterior.

The MRI analysis showed typical focal lesions in the CA1 region of the cornu ammonis (67% unilateral, 19% bilateral) (Table 1). In three subjects, no lesion could be detected. In 29% of patients, the lesion was located in the ventral hippocampus, in 52% in the intermediate and in 24% in the dorsal hippocampus (Figures 3, 4). There was no significant association between the position of lesions and age (left/right: *r* = −0.07, *p* = 0.79, ventral/dorsal: *r* = −0.53, *p* = 0.18), sex (left/right: χ^2^ = 0.78, *df* = 1, Fisher’s exact test = 0.60, ventral/dorsal: χ^2^ = 0.95, *df* = 3, *p* = 0.81), stress related factors (left/right: PSS: *r* = −0.7, *p* = 0.55, HADS-A: *r* = −0.07, *p* = 0.79, HADS-D: *r* = 0.22, *p* = 0.42, SRRS: *r* = 0.38, *p* = 0.15, vent/dor: PSS: *r* = 0.67, *p* = 0.07, HADS-A: *r* = −0.31, *p* = 0.46, HADS-D: *r* = −0.13, *p* = 0.76, SRRS: *r* = 0.02, *p* = 0.96) and precipitating events (left/right: χ^2^ = 3.31, *df* = 1, Fisher’s exact test = 0.12, ventral/dorsal: χ^2^ = 4.39, *df* = 3, *p* = 0.22).

**Table 1:** **Epidemiological and imaging data of TGA patients with a stress-related and physical precipitant event**.

Pat.ID	Sex	Age	Precipitating events	Position of hippocampal lesions
**Stress-related**				
S1	F	74	Son had brain surgery that day	Left-ant
S2	F	71	Emotional arousal after quarrel	none
S3	F	65	First encounter with new-born grandchild	Left-ant, right-mid
S4	M	71	Discharge from hospital with diagnosis of cancer	Left-ant
S5	F	67	Fear for sick child	Left-ant
S6	F	65	Emotional stress with sick husband	Left-mid
S7	M	69	Acute marital conflict	None
S8	F	65	Emotional Stress with mother	Left-mid, right-post
**Physical**				
P1	F	66	After cycling	Left-mid, left-post
P2	F	61	Postcoital	Left-mid
P3	F	66	Swimming in a lake	Right- mid
P4	M	67	Snow- shoveling	Right- mid
P5	M	67	During cycling	Left-mid, right-mid
P6	M	66	Postcoital	Right-post
P7	M	68	After renovation work	Left-mid
P8	F	69	After heavy lifting	Right-mid
P9	F	62	Heavy gardening	Left-post
P10	M	70	After work on a boat	Left-mid
P11	M	57	After weight lifting	Left-post, right-ant
P12	M	57	After chain sawing	Right-ant
P13	M	50	After weight lifting	none

## Discussion

Our results show that about a fifth of patients experienced a stress related precipitant event immediately before an acute TGA episode. In patients who experienced an emotional precipitating event a significant higher anxiety level was observed. Additionally, TGA patients differed from healthy controls in certain aspects of coping behavior. Taken together, our findings indicate a role of stress in TGA manifestation in a subgroup of patients but no direct association between precipitant stress episodes and the emergence of CA1 lesions. We could not detect a clear functional differentiation of hippocampal anatomy into mnemonic and stress-related compartments.

### Precipitant events and stress

In the clinical study of TGA, the incidence of events directly preceding a TGA has been of great interest as the etiology of TGA decades after its first clinical description remains cryptic (Fisher and Adams, 1964; Quinette et al., 2006). Typically, precipitating events that include physical or emotional episodes can be detected in the majority of patients. In terms of different classes (emotional stress, physical effort, acute pain, water contact, temperature change, sexual intercourse etc.), the distribution of precipitating events in our large cohort is comparable to the observation of Quinette et al. (2006). Based on a hierarchical cluster analysis, they identified three distinct groups of TGA patients: a physical precipitating event in men, younger patients with a history of migraine and women with an emotional precipitant. Relating to the latter cluster, we could show a significant association between an elevated level of anxiety preceding the attack and the appearance of an emotionally induced TGA. Two significant differences in coping strategies between controls and patients show that controls tend to downplay their troubles by comparing with other people, whereas patients experience more often feelings of guilt. This poses the hypothesis that TGA patients tend to possess less advantageous strategies in handling of stress than healthy controls. In stressful situations, TGA patients apparently focus on internal processes, whereas the controls tend to regulatory strategies maintaining the self-esteem by beneficial social comparisons and distraction. Considering this, coping and stress are directly related to each other: inadequate strategies impair the emotional adjustment and other intrinsic factors that affect the individual stress response. Within a neurobiological framework, it has been suggested that the individual variability in the adaptation and coping to stress is dependent on the interaction between genetic and cognitive/emotional factors in which glucocorticoid hormones and receptors play a critical role (Oitzl et al., 2010). Depressed mood and a high number of stress inducing life events in the past two years before the TGA (measured by the SRRS), however, exerted no influence on precipitant events. However, as a limitation of our study, we cannot rule out, that the patient sample size was not large enough to reach significance with the other stress-related measures. Alternatively, the link between stress, hippocampal (CA1) dysfunction and memory disorders might be an association between more complex functional, neuroendocrine and genetical factors (Schwabe et al., 2012; Wingenfeld and Wolf, 2014). Studies with larger sample sizes are necessary to extend our findings in the future.

### Transient global amnesia and emotional factors

In recent years, the association of TGA with psychopathological factors became a greater focus of attention emphasizing the role of psychological factors in TGA (Merriam et al., 1992; Inzitari et al., 1997; Pantoni et al., 2005; Noël et al., 2007, 2008, 2011). In their large case series, Quinette et al. (2006) observed an association between the occurrence of a TGA in women, a history of anxiety and a pathological personality. About a third of that sample was found to have a personality disorder, an anxious depressive profile or emotional instability. Our data corroborate their finding of a particular sensitivity to psychological stress in TGA patients. Similarly, Pantoni et al. (2005) observed a higher prevalence of a personal or family history of psychiatric conditions and diseases and phobic traits.

The most frequently studied stress related concepts in connection with TGA are anxiety and depression. Neri et al. found a negative association between retrograde amnesia and depression (Neri et al., 1995). Noël et al. (2007) suggest that high levels of anxiety and depression can aggravate the deficits in episodic memory and might have a detrimental influence on the recovery process of TGA. In terms of anxiety, a more specific investigation was published by Inzitari et al. (1997). They examined selective personality traits (agoraphobic and simple phobia attitudes) as possible predisposing factors for TGA, compared to controls with a TIA. Both, agoraphobic and simple phobia attitudes were significantly higher amongst patients with TGA. Additionally, many patients in this study had symptoms resembling a panic attack during TGA. Pantoni et al. (2005) conceptualize the involvement of the limbic regions in anxiety and emotion with the hypothesis of a local CBF decrease in patients during panic attacks. Other concepts complement the idea of an association between a predispositional vulnerability and diathesis with functional neuronal changes during a precipitating event. The coincidence of these factors may lead to a cascade of stress induced release of steroid stress mediators and glutamatergic neurotransmitters eliciting cytotoxicity in the hippocampus (Quinette et al., 2006; Joëls et al., 2008; Bartsch and Deuschl, 2010). Kessler et al. also suggested a major role of psychological stress and the release of stress hormones in both, the etiology and the recovery process of TGA (Kessler et al., 2001). Future studies should therefore focus on the characterization of the neuroendocrine response in TGA (de Kloet et al., 2012), a clear limitation of our study. Our data fit into the aforementioned concepts of TGA being a result of a cascade with a stress related trigger in a subgroup of patients. From a mechanistic viewpoint, several triggers including stress responses may therefore promote the final pathophysiological pathway in TGA with a functional and structural dysfunction of CA1. The CA1 region of hippocampus is selectively vulnerable to a variety of metabolic and excitotoxic insults which can be most prominently seen in acute neurological conditions such as hypoxia-ischemia, hypoglycemia and epilepsy. In TGA, hippocampal CA1 lesions as seen on MRI are a reflection of signal changes due to impaired intracellular diffusion and cytotoxic edema in the affected region. Thus, the lesions most likely emerge as a results of a noxious input affecting the cellular metabolism in hippocampal neurons (Bartsch et al., 2008). The basis of this regional susceptibility is, however, poorly understood (Michaelis, 2012). Under consideration of this regional vulnerability, the question arises, whether acute psychological stress can directly facilitate the emergence of hippocampal CA1 lesions. Further future research directions should also include the impact of stress on the structural integrity of CA1 neurons and an evaluation of the genetic background of hippocampal vulnerability (Schmidt et al., 2008; Papassotiropoulos and de Quervain, 2011; Molendijk et al., 2012). With regard to the neuropsychological deficit in TGA, recent experimental studies suggest that local dysfunction in hippocampal CA1 neurons may lead to a transient perturbation of hippocampal function (Cohen et al., 2013). Alternatively, lesions in TGA may lead to in a diachisis of memory function in widespread hippocampal networks (Peer et al., 2014).

### Stress and the hippocampus

The concept of stress is defined as a multidimensional construct consisting of “(i) stress input with perception and appraisal of the stressor, (ii) the processing of stressful information and (iii) the stress response itself with the objective of restoring homeostasis through behavioral and physiological adaptations” (de Kloet, 2012). The hippocampus with its CA1 neurons is particularly integrated in the stress responses to emotional stressors including fear and anxiety (Howland and Wang, 2008; Joëls et al., 2008; Joëls, 2009). The hippocampus has a reciprocal involvement in the regulation of stress by influence on the hypothalamic-pituitary-adrenal axis, which in turn affects the hippocampus by the release of stress hormones (de Kloet et al., 2005). Harmful effects of stress are thought to lead to various cellular and systemic effects in terms of changes in synaptic plasticity and excitability, hippocampal neurogenesis, structural changes and memory deficits (McEwen and Milner, 2007; Sandi and Pinelo-Nava, 2007; de Kloet, 2012). In animals, it has repetitively been shown that acute behavioral stress leads to a reduction of hippocampal long term potentiation (LTP) and an enhancement of long term depression (LTD) in CA1 (Howland and Wang, 2008). Similarly, stress enhanced glutamatergic transmission leads to an increase of calcium influx in CA1 neurons and in succession to a metabolic vulnerability and, under certain circumstances, to an impairment of structural integrity (Joëls, 2009).

Recently, a functional compartmentalization of the hippocampus based on gene expression in animals has been suggested of the existence of a confined, dorsal “cognitive hippocampus” as a critical relay unit for learning and memory processes. Complementary, the concept of a ventral “emotional” hippocampus suggests that this compartment is involved in emotional, affective and stress regulation. An intermediate zone of this structure is thought to integrate cognitive and spatial processing into behavioral relevant actions (Gray and McNaughton, 1983; Moser and Moser, 1998; Fanselow and Dong, 2010; Maggio and Segal, 2012). Our results in TGA as natural lesion model of hippocampal function, correlating hippocampal lesions with cognitive and behavioral deficits as well as stress-related factors, however, did not show evidence for a similar longitudinal unitization existing in humans. Further research should therefore evaluate the concept of a functional differentiation of the hippocampus in humans using functional imaging.

## Author contributions

Juliane Döhring designed the experiment, performed the analysis, interpreted the results, drafted and revised the manuscript. Alexander Schmuck recruited the participants, performed data acquisition and revised the MS. Thorsten Bartsch designed the experiment, performed data acquisition, interpreted the data, drafted and revised the manuscript.

## Conflict of interest statement

The authors declare that the research was conducted in the absence of any commercial or financial relationships that could be construed as a potential conflict of interest.
